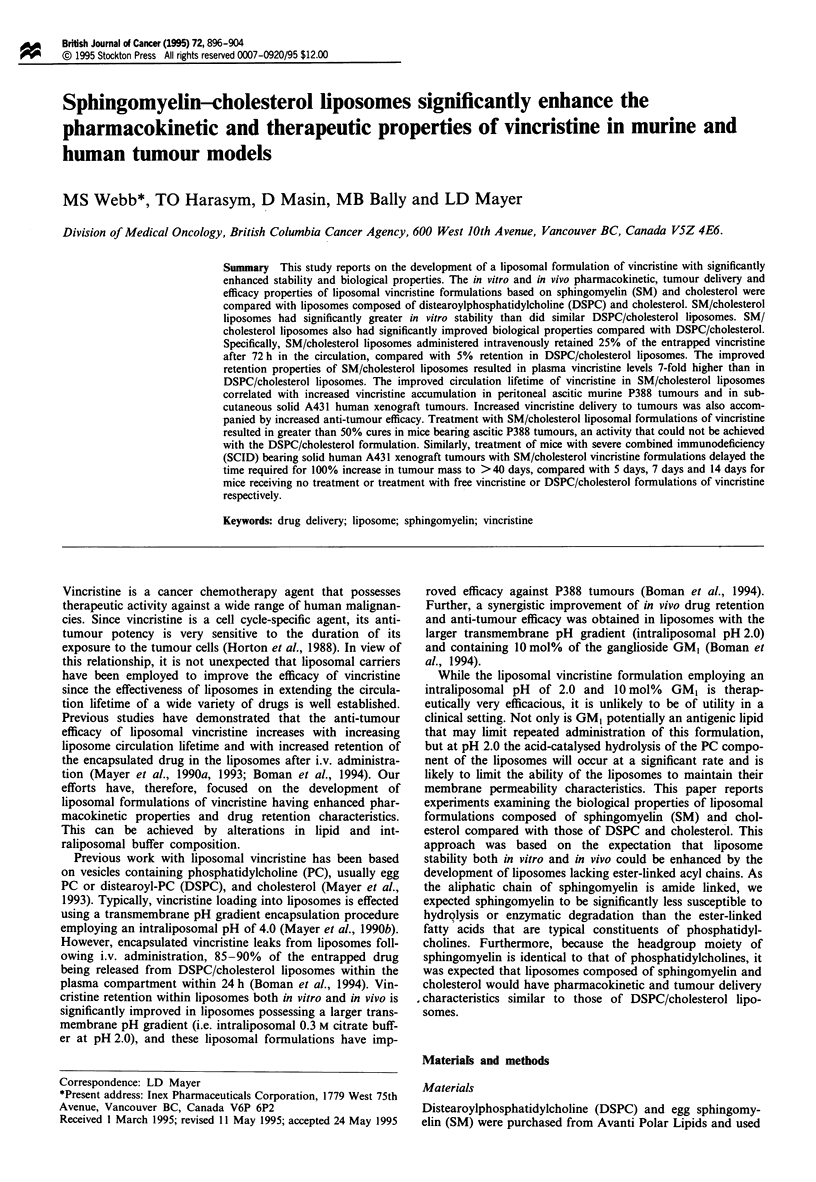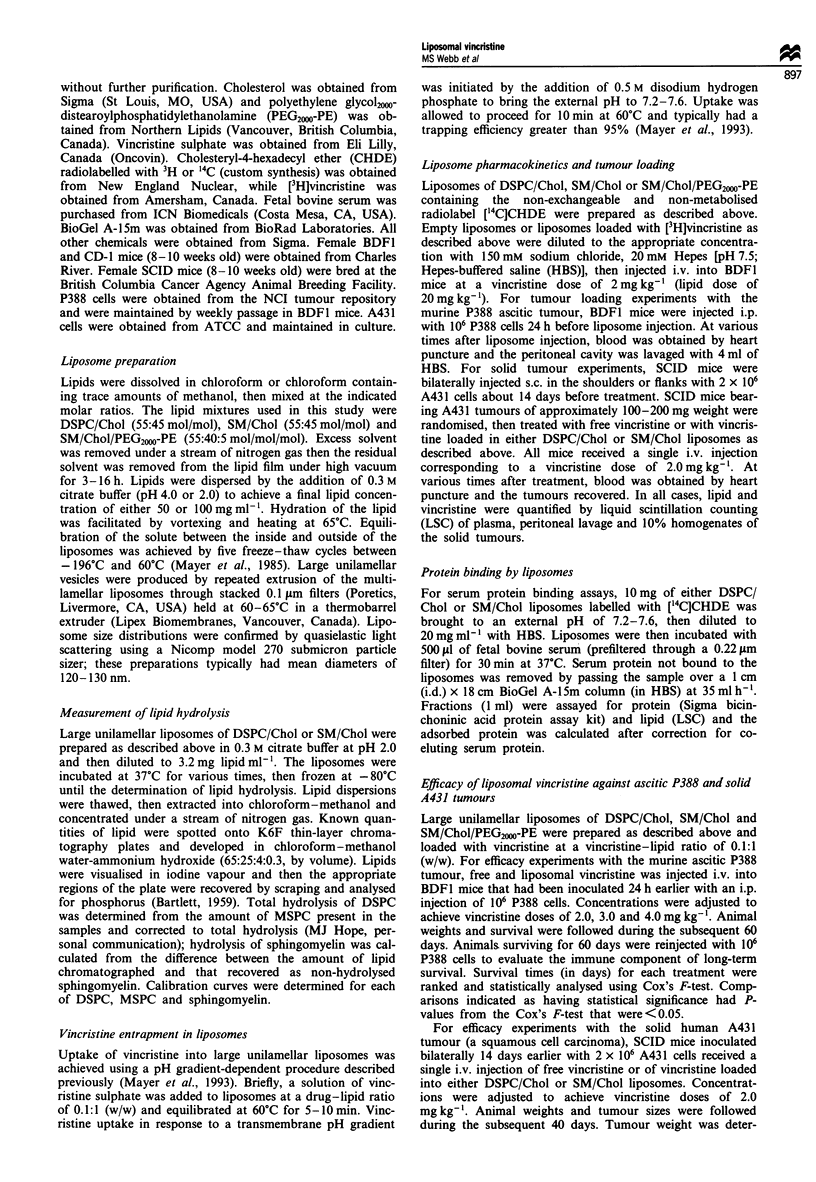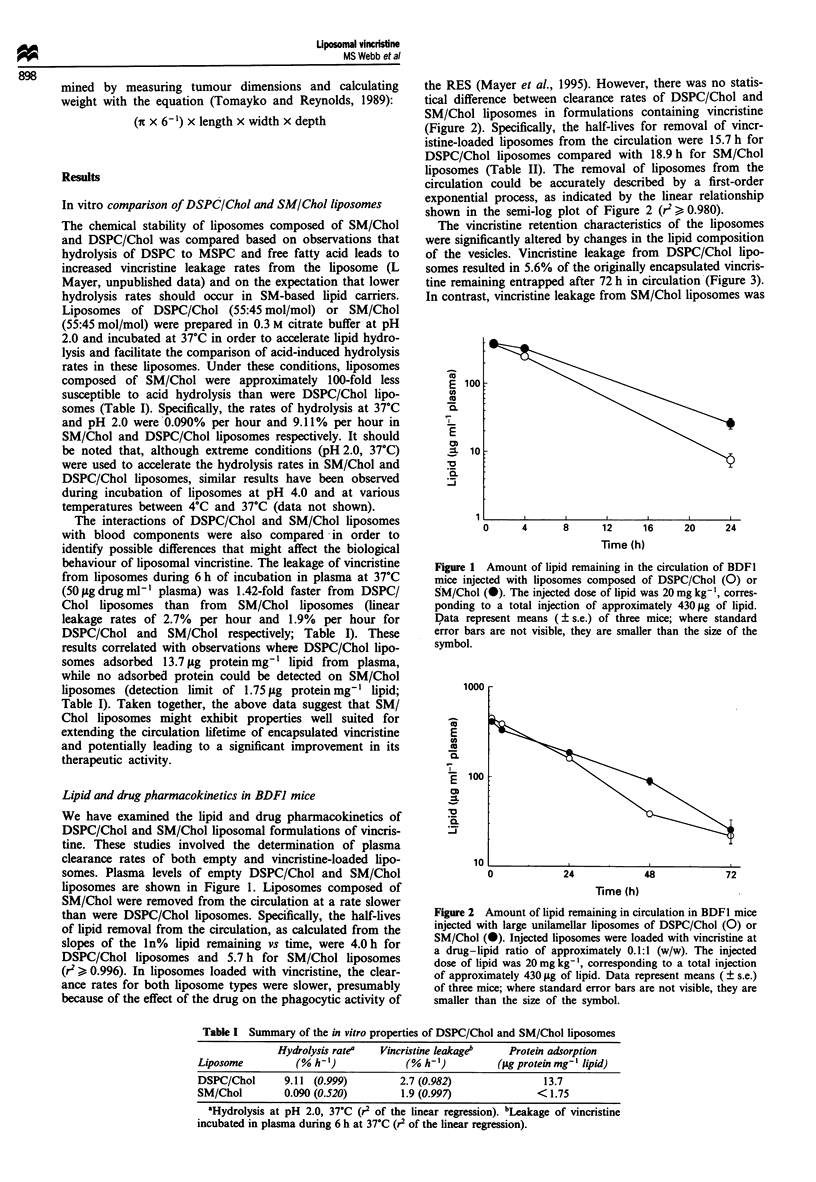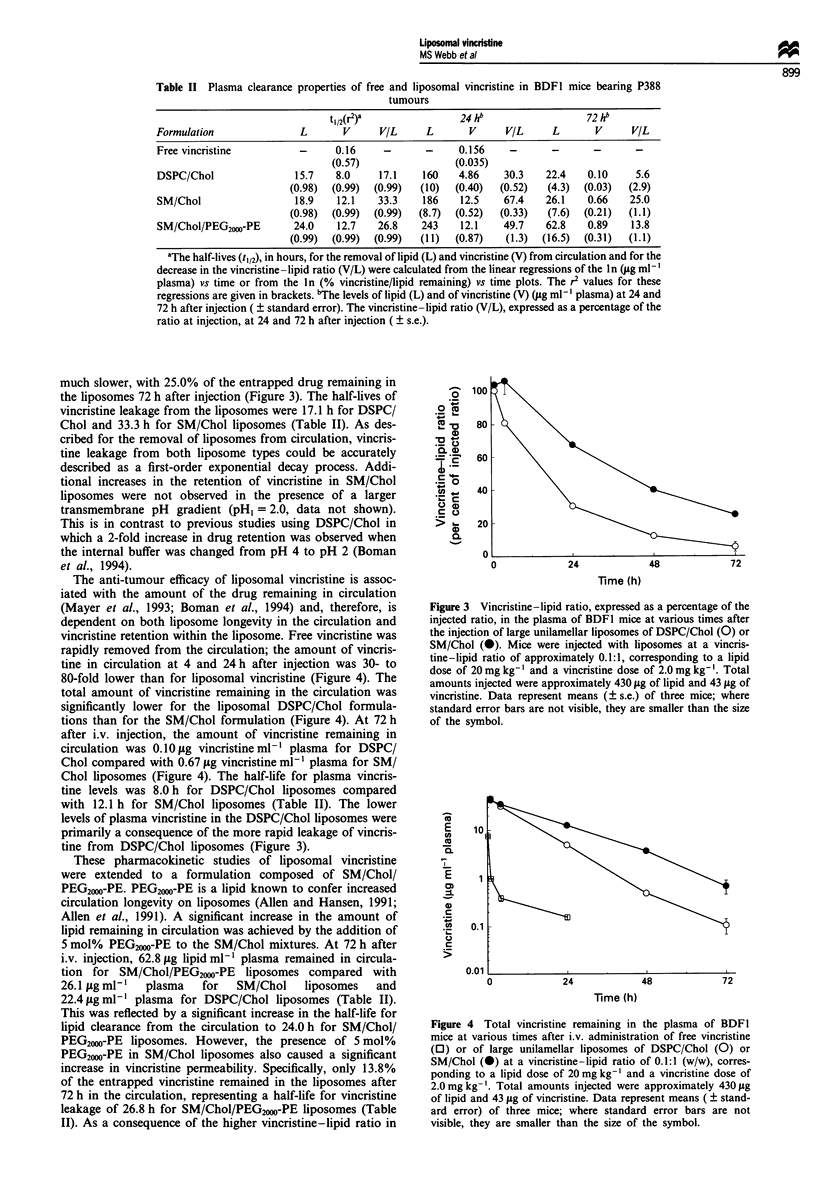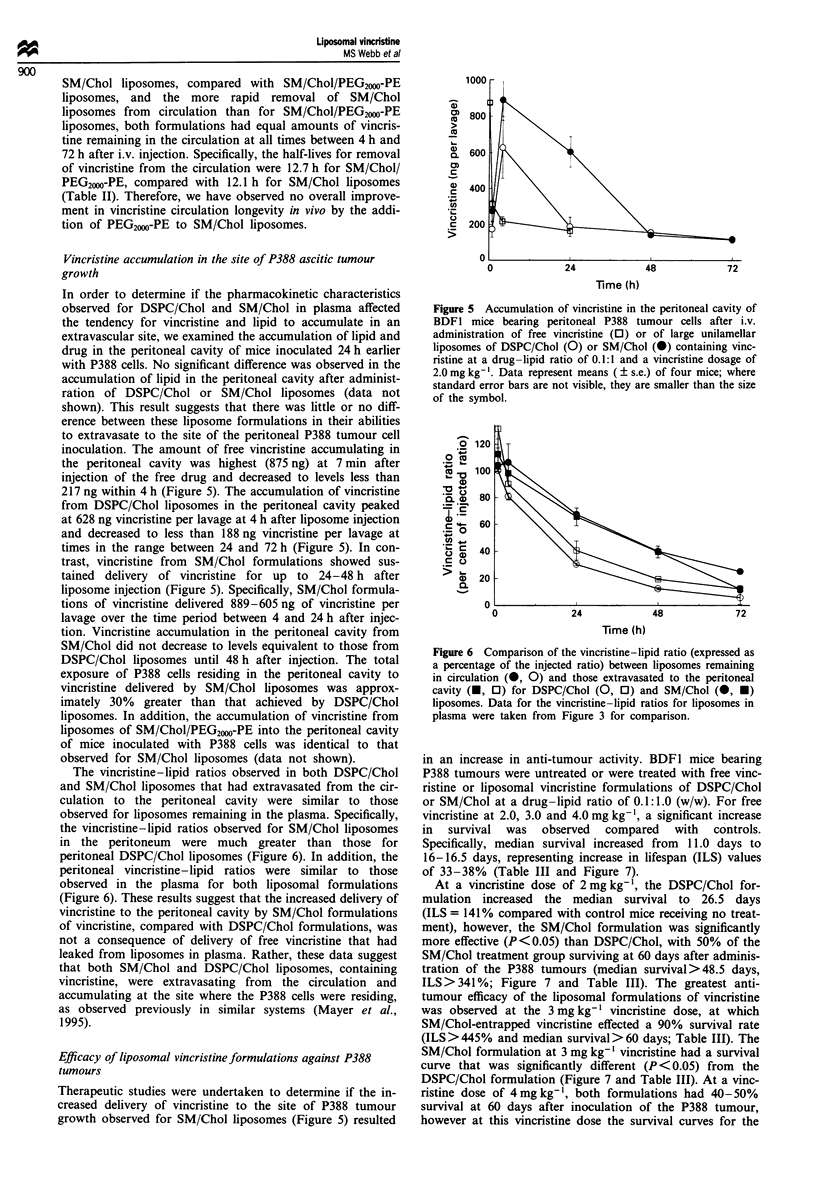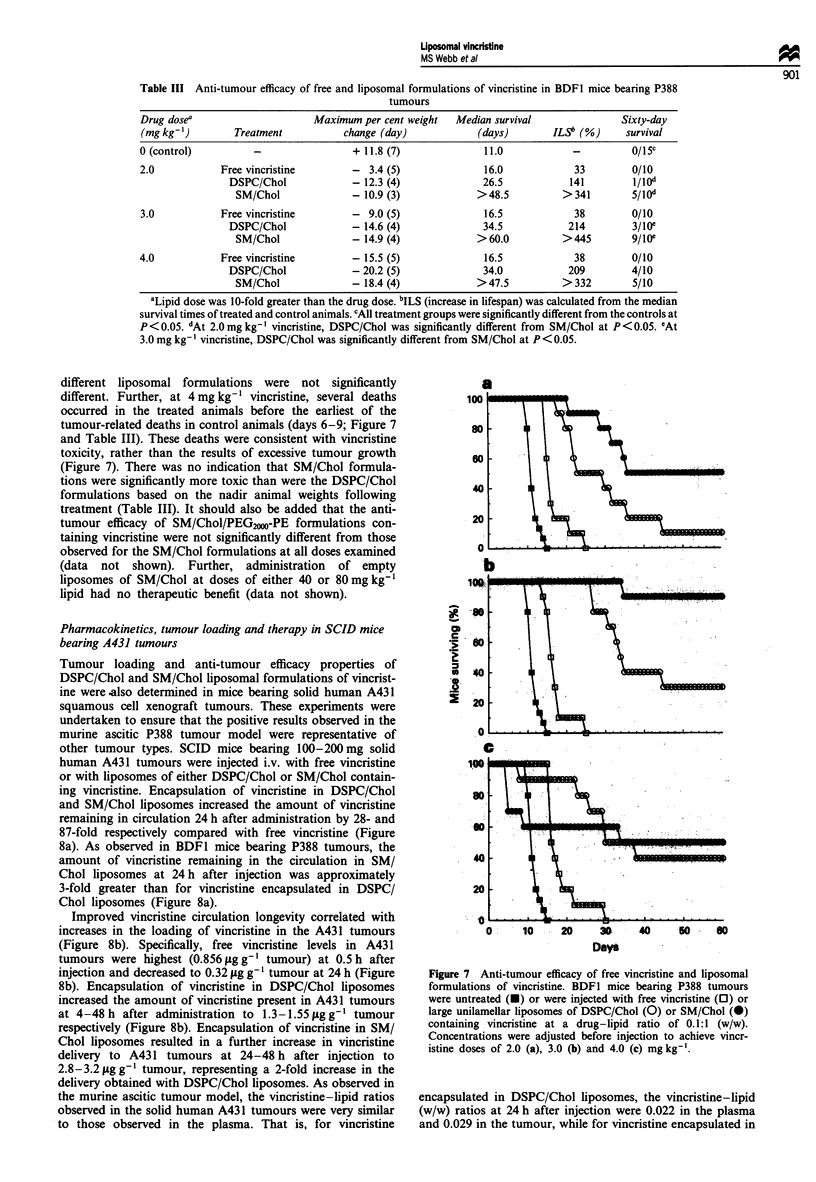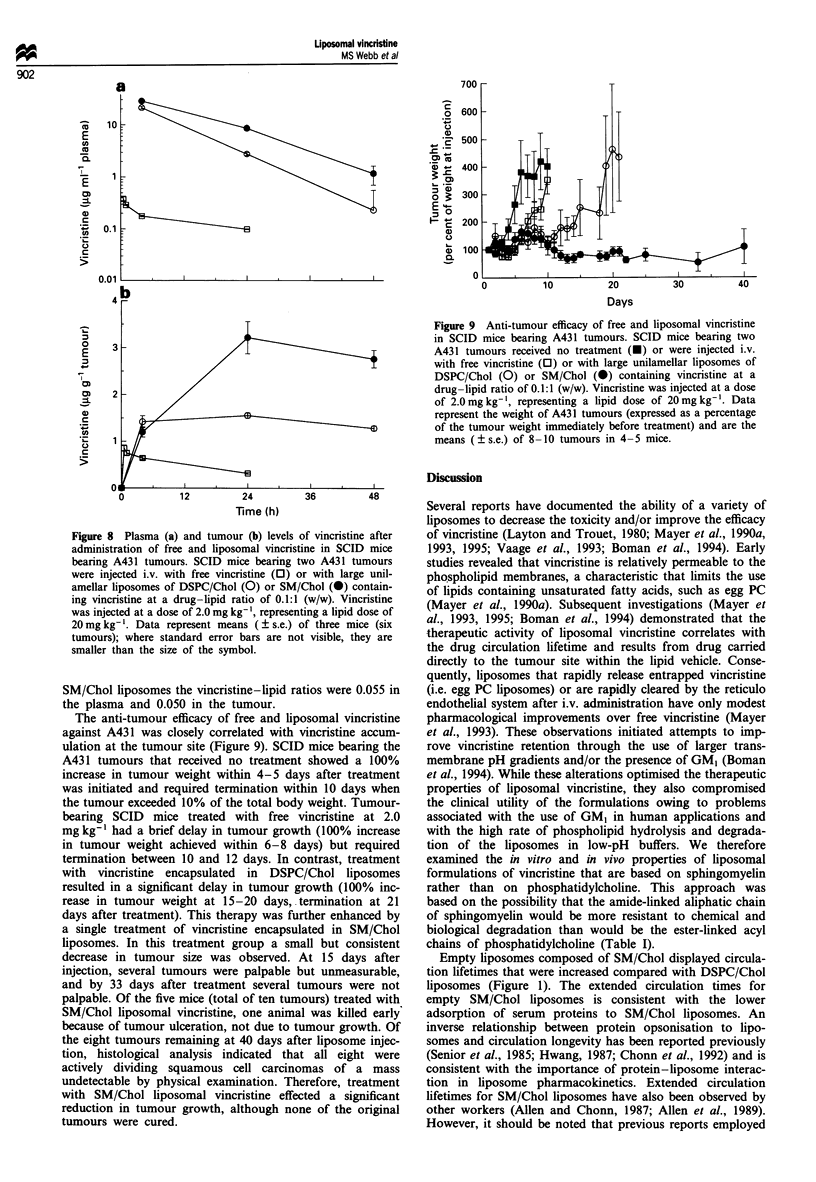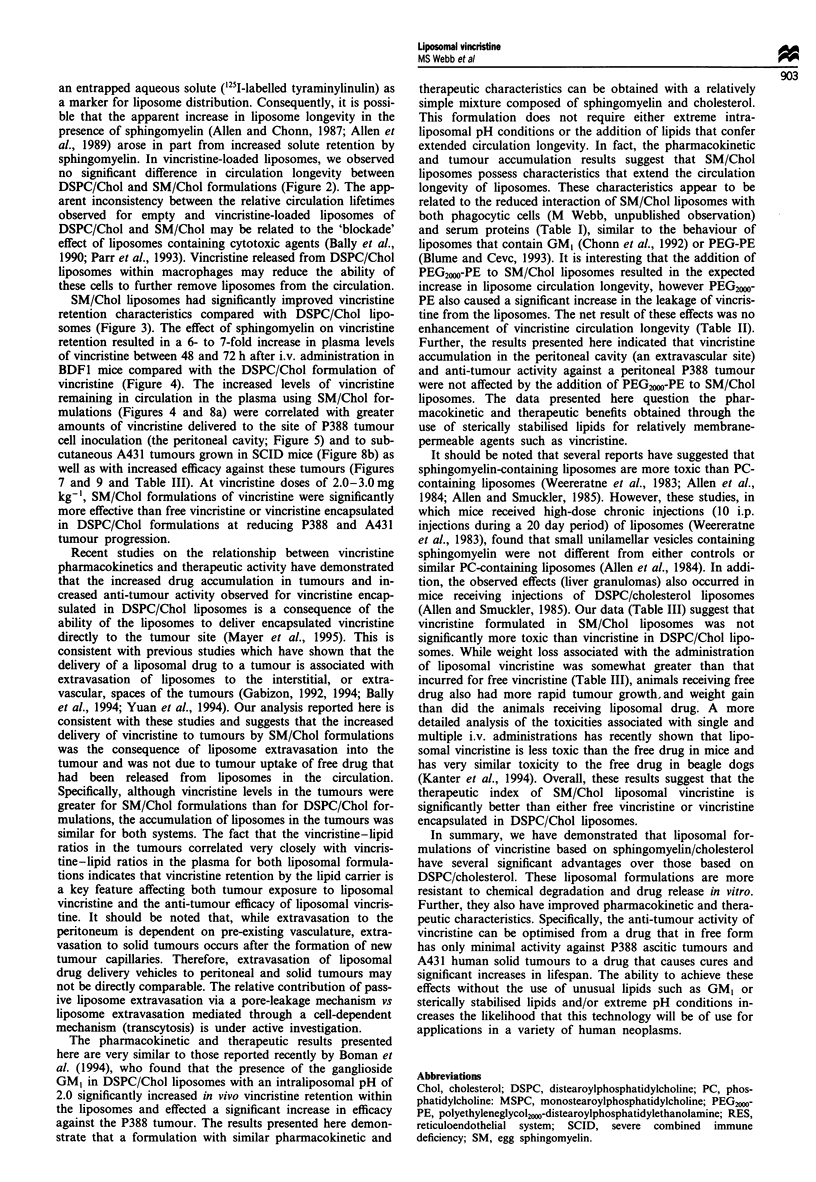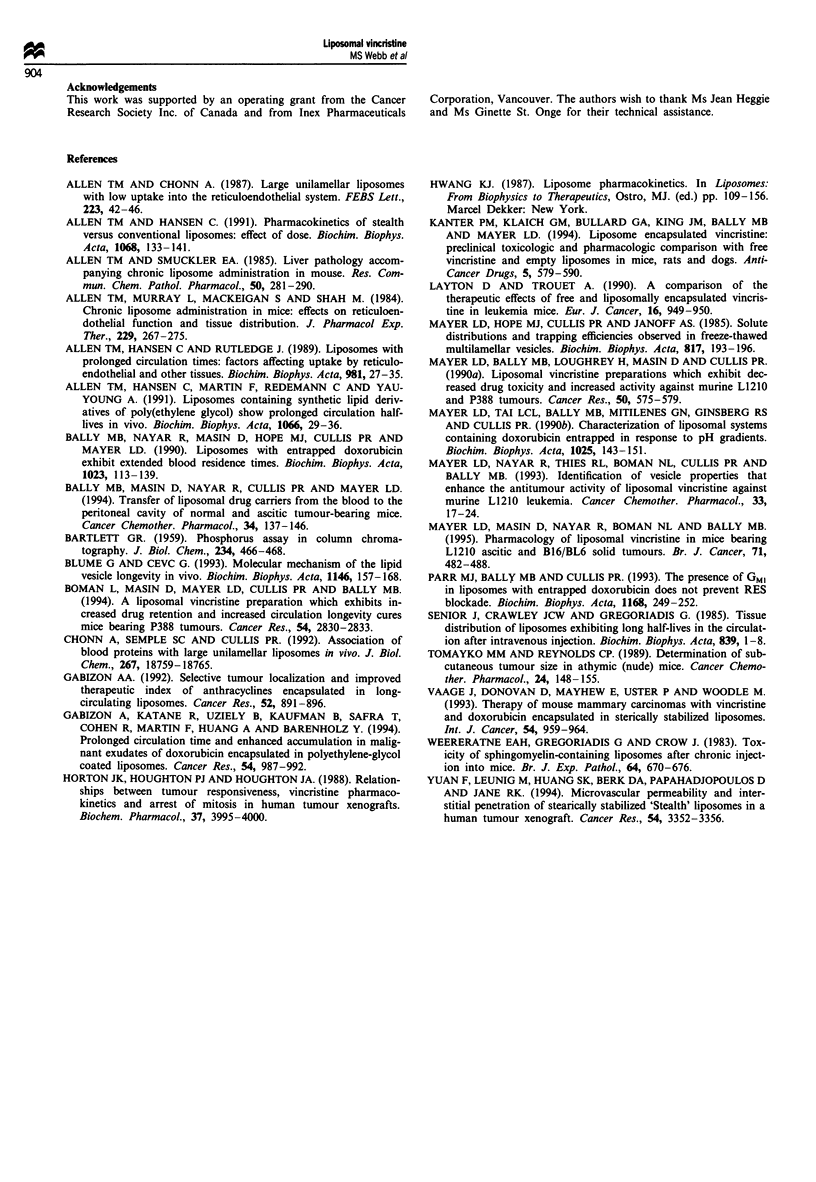# Sphingomyelin-cholesterol liposomes significantly enhance the pharmacokinetic and therapeutic properties of vincristine in murine and human tumour models.

**DOI:** 10.1038/bjc.1995.430

**Published:** 1995-10

**Authors:** M. S. Webb, T. O. Harasym, D. Masin, M. B. Bally, L. D. Mayer

**Affiliations:** Division of Medical Oncology, British Columbia Cancer Agency, Vancouver, Canada.

## Abstract

This study reports on the development of a liposomal formulation of vincristine with significantly enhanced stability and biological properties. The in vitro and in vivo pharmacokinetic, tumour delivery and efficacy properties of liposomal vincristine formulations based on sphingomyelin (SM) and cholesterol were compared with liposomes composed of distearoylphosphatidylcholine (DSPC) and cholesterol. SM/cholesterol liposomes had significantly greater in vitro stability than did similar DSPC/cholesterol liposomes. SM/cholesterol liposomes also had significantly improved biological properties compared with DSPC/cholesterol. Specifically, SM/cholesterol liposomes administered intravenously retained 25% of the entrapped vincristine after 72 h in the circulation, compared with 5% retention in DSPC/cholesterol liposomes. The improved retention properties of SM/cholesterol liposomes resulted in plasma vincristine levels 7-fold higher than in DSPC/cholesterol liposomes. The improved circulation lifetime of vincristine in SM/cholesterol liposomes correlated with increased vincristine accumulation in peritoneal ascitic murine P388 tumours and in subcutaneous solid A431 human xenograft tumours. Increased vincristine delivery to tumours was also accompanied by increased anti-tumour efficacy. Treatment with SM/cholesterol liposomal formulations of vincristine resulted in greater than 50% cures in mice bearing ascitic P388 tumours, an activity that could not be achieved with the DSPC/cholesterol formulation. Similarly, treatment of mice with severe combined immunodeficiency (SCID) bearing solid human A431 xenograft tumours with SM/cholesterol vincristine formulations delayed the time required for 100% increase in tumour mass to > 40 days, compared with 5 days, 7 days and 14 days for mice receiving no treatment or treatment with free vincristine or DSPC/cholesterol formulations of vincristine respectively.


British Journal of Cancer (1995) 72, 896-904
© 1995 Stockton Press All rights reserved 0007-0920/95 $12.00

### Sphingomyelin–cholesterol liposomes significantly enhance the pharmacokinetic and therapeutic properties of vincristine in murine and human tumour models

MS Webb*, TO Harasym, D Masin, MB Bally and LD Mayer

*Division of Medical Oncology, British Columbia Cancer Agency, 600 West 10th Avenue, Vancouver BC, Canada V5Z 4E6.*

**Summary** This study reports on the development of a liposomal formulation of vincristine with significantly enhanced stability and biological properties. The *in vitro* and *in vivo* pharmacokinetic, tumour delivery and efficacy properties of liposomal vincristine formulations based on sphingomyelin (SM) and cholesterol were compared with liposomes composed of distearoylphosphatidylcholine (DSPC) and cholesterol. SM/cholesterol liposomes had significantly greater *in vitro* stability than did similar DSPC/cholesterol liposomes. SM/cholesterol liposomes also had significantly improved biological properties compared with DSPC/cholesterol. Specifically, SM/cholesterol liposomes administered intravenously retained 25% of the entrapped vincristine after 72 h in the circulation, compared with 5% retention in DSPC/cholesterol liposomes. The improved retention properties of SM/cholesterol liposomes resulted in plasma vincristine levels 7-fold higher than in DSPC/cholesterol liposomes. The improved circulation lifetime of vincristine in SM/cholesterol liposomes correlated with increased vincristine accumulation in peritoneal ascitic murine P388 tumours and in subcutaneous solid A431 human xenograft tumours. Increased vincristine delivery to tumours was also accompanied by increased anti-tumour efficacy. Treatment with SM/cholesterol liposomal formulations of vincristine resulted in greater than 50% cures in mice bearing ascitic P388 tumours, an activity that could not be achieved with the DSPC/cholesterol formulation. Similarly, treatment of mice with severe combined immunodeficiency (SCID) bearing solid human A431 xenograft tumours with SM/cholesterol vincristine formulations delayed the time required for 100% increase in tumour mass to >40 days, compared with 5 days, 7 days and 14 days for mice receiving no treatment or treatment with free vincristine or DSPC/cholesterol formulations of vincristine respectively.

**Keywords:** drug delivery; liposome; sphingomyelin; vincristine

Vincristine is a cancer chemotherapy agent that possesses therapeutic activity against a wide range of human malignancies. Since vincristine is a cell cycle-specific agent, its anti-tumour potency is very sensitive to the duration of its exposure to the tumour cells (Horton *et al*., 1988). In view of this relationship, it is not unexpected that liposomal carriers have been employed to improve the efficacy of vincristine since the effectiveness of liposomes in extending the circulation lifetime of a wide variety of drugs is well established. Previous studies have demonstrated that the anti-tumour efficacy of liposomal vincristine increases with increasing liposome circulation lifetime and with increased retention of the encapsulated drug in the liposomes after i.v. administration (Mayer *et al*., 1990*a*, 1993; Boman *et al*., 1994). Our efforts have, therefore, focused on the development of liposomal formulations of vincristine having enhanced pharmacokinetic properties and drug retention characteristics. This can be achieved by alterations in lipid and intraliposomal buffer composition.

Previous work with liposomal vincristine has been based on vesicles containing phosphatidylcholine (PC), usually egg PC or distearoyl-PC (DSPC), and cholesterol (Mayer *et al*., 1993). Typically, vincristine loading into liposomes is effected using a transmembrane pH gradient encapsulation procedure employing an intraliposomal pH of 4.0 (Mayer *et al*., 1990*b*). However, encapsulated vincristine leaks from liposomes following i.v. administration, 85–90% of the entrapped drug being released from DSPC/cholesterol liposomes within the plasma compartment within 24 h (Boman *et al*., 1994). Vincristine retention within liposomes both *in vitro* and *in vivo* is significantly improved in liposomes possessing a larger transmembrane pH gradient (i.e. intraliposomal 0.3 M citrate buffer at pH 2.0), and these liposomal formulations have improved efficacy against P388 tumours (Boman *et al*., 1994). Further, a synergistic improvement of *in vivo* drug retention and anti-tumour efficacy was obtained in liposomes with the larger transmembrane pH gradient (intraliposomal pH 2.0) and containing 10 mol% of the ganglioside $GM_1$ (Boman *et al*., 1994).

While the liposomal vincristine formulation employing an intraliposomal pH of 2.0 and 10 mol% $GM_1$ is therapeutically very efficacious, it is unlikely to be of utility in a clinical setting. Not only is $GM_1$ potentially an antigenic lipid that may limit repeated administration of this formulation, but at pH 2.0 the acid-catalysed hydrolysis of the PC component of the liposomes will occur at a significant rate and is likely to limit the ability of the liposomes to maintain their membrane permeability characteristics. This paper reports experiments examining the biological properties of liposomal formulations composed of sphingomyelin (SM) and cholesterol compared with those of DSPC and cholesterol. This approach was based on the expectation that liposome stability both *in vitro* and *in vivo* could be enhanced by the development of liposomes lacking ester-linked acyl chains. As the aliphatic chain of sphingomyelin is amide linked, we expected sphingomyelin to be significantly less susceptible to hydrolysis or enzymatic degradation than the ester-linked fatty acids that are typical constituents of phosphatidylcholines. Furthermore, because the headgroup moiety of sphingomyelin is identical to that of phosphatidylcholines, it was expected that liposomes composed of sphingomyelin and cholesterol would have pharmacokinetic and tumour delivery characteristics similar to those of DSPC/cholesterol liposomes.

### Materials and methods

### *Materials*

Distearoylphosphatidylcholine (DSPC) and egg sphingomyelin (SM) were purchased from Avanti Polar Lipids and used

Correspondence: LD Mayer
*Present address: Inex Pharmaceuticals Corporation, 1779 West 75th Avenue, Vancouver BC, Canada V6P 6P2
Received 1 March 1995; revised 11 May 1995; accepted 24 May 1995

without further purification. Cholesterol was obtained from Sigma (St Louis, MO, USA) and polyethylene glycol$_{2000}$-distearoylphosphatidylethanolamine (PEG$_{2000}$-PE) was obtained from Northern Lipids (Vancouver, British Columbia, Canada). Vincristine sulphate was obtained from Eli Lilly, Canada (Oncovin). Cholesteryl-4-hexadecyl ether (CHDE) radiolabelled with $^{3}$H or $^{14}$C (custom synthesis) was obtained from New England Nuclear, while [$^{3}$H]vincristine was obtained from Amersham, Canada. Fetal bovine serum was purchased from ICN Biomedicals (Costa Mesa, CA, USA). BioGel A-15m was obtained from BioRad Laboratories. All other chemicals were obtained from Sigma. Female BDF1 and CD-1 mice (8–10 weeks old) were obtained from Charles River. Female SCID mice (8–10 weeks old) were bred at the British Columbia Cancer Agency Animal Breeding Facility. P388 cells were obtained from the NCI tumour repository and were maintained by weekly passage in BDF1 mice. A431 cells were obtained from ATCC and maintained in culture.

*Liposome preparation*

Lipids were dissolved in chloroform or chloroform containing trace amounts of methanol, then mixed at the indicated molar ratios. The lipid mixtures used in this study were DSPC/Chol (55:45 mol/mol), SM/Chol (55:45 mol/mol) and SM/Chol/PEG$_{2000}$-PE (55:40:5 mol/mol/mol). Excess solvent was removed under a stream of nitrogen gas then the residual solvent was removed from the lipid film under high vacuum for 3–16 h. Lipids were dispersed by the addition of 0.3 M citrate buffer (pH 4.0 or 2.0) to achieve a final lipid concentration of either 50 or 100 mg ml$^{-1}$. Hydration of the lipid was facilitated by vortexing and heating at 65°C. Equilibration of the solute between the inside and outside of the liposomes was achieved by five freeze–thaw cycles between −196°C and 60°C (Mayer *et al*., 1985). Large unilamellar vesicles were produced by repeated extrusion of the multilamellar liposomes through stacked 0.1 μm filters (Poretics, Livermore, CA, USA) held at 60–65°C in a thermobarrel extruder (Lipex Biomembranes, Vancouver, Canada). Liposome size distributions were confirmed by quasielastic light scattering using a Nicomp model 270 submicron particle sizer; these preparations typically had mean diameters of 120–130 nm.

*Measurement of lipid hydrolysis*

Large unilamellar liposomes of DSPC/Chol or SM/Chol were prepared as described above in 0.3 M citrate buffer at pH 2.0 and then diluted to 3.2 mg lipid ml$^{-1}$. The liposomes were incubated at 37°C for various times, then frozen at −80°C until the determination of lipid hydrolysis. Lipid dispersions were thawed, then extracted into chloroform–methanol and concentrated under a stream of nitrogen gas. Known quantities of lipid were spotted onto K6F thin-layer chromatography plates and developed in chloroform–methanol water-ammonium hydroxide (65:25:4:0.3, by volume). Lipids were visualised in iodine vapour and then the appropriate regions of the plate were recovered by scraping and analysed for phosphorus (Bartlett, 1959). Total hydrolysis of DSPC was determined from the amount of MSPC present in the samples and corrected to total hydrolysis (MJ Hope, personal communication); hydrolysis of sphingomyelin was calculated from the difference between the amount of lipid chromatographed and that recovered as non-hydrolysed sphingomyelin. Calibration curves were determined for each of DSPC, MSPC and sphingomyelin.

*Vincristine entrapment in liposomes*

Uptake of vincristine into large unilamellar liposomes was achieved using a pH gradient-dependent procedure described previously (Mayer *et al*., 1993). Briefly, a solution of vincristine sulphate was added to liposomes at a drug–lipid ratio of 0.1:1 (w/w) and equilibrated at 60°C for 5–10 min. Vincristine uptake in response to a transmembrane pH gradient was initiated by the addition of 0.5 M disodium hydrogen phosphate to bring the external pH to 7.2–7.6. Uptake was allowed to proceed for 10 min at 60°C and typically had a trapping efficiency greater than 95% (Mayer *et al*., 1993).

*Liposome pharmacokinetics and tumour loading*

Liposomes of DSPC/Chol, SM/Chol or SM/Chol/PEG$_{2000}$-PE containing the non-exchangeable and non-metabolised radiolabel [$^{14}$C]CHDE were prepared as described above. Empty liposomes or liposomes loaded with [$^{3}$H]vincristine as described above were diluted to the appropriate concentration with 150 mM sodium chloride, 20 mM Hepes [pH 7.5; Hepes-buffered saline (HBS)], then injected i.v. into BDF1 mice at a vincristine dose of 2 mg kg$^{-1}$ (lipid dose of 20 mg kg$^{-1}$). For tumour loading experiments with the murine P388 ascitic tumour, BDF1 mice were injected i.p. with 10$^{6}$ P388 cells 24 h before liposome injection. At various times after liposome injection, blood was obtained by heart puncture and the peritoneal cavity was lavaged with 4 ml of HBS. For solid tumour experiments, SCID mice were bilaterally injected s.c. in the shoulders or flanks with 2 × 10$^{6}$ A431 cells about 14 days before treatment. SCID mice bearing A431 tumours of approximately 100–200 mg weight were randomised, then treated with free vincristine or with vincristine loaded in either DSPC/Chol or SM/Chol liposomes as described above. All mice received a single i.v. injection corresponding to a vincristine dose of 2.0 mg kg$^{-1}$. At various times after treatment, blood was obtained by heart puncture and the tumours recovered. In all cases, lipid and vincristine were quantified by liquid scintillation counting (LSC) of plasma, peritoneal lavage and 10% homogenates of the solid tumours.

*Protein binding by liposomes*

For serum protein binding assays, 10 mg of either DSPC/Chol or SM/Chol liposomes labelled with [$^{14}$C]CHDE was brought to an external pH of 7.2–7.6, then diluted to 20 mg ml$^{-1}$ with HBS. Liposomes were then incubated with 500 μl of fetal bovine serum (prefiltered through a 0.22 μm filter) for 30 min at 37°C. Serum protein not bound to the liposomes was removed by passing the sample over a 1 cm (i.d.) × 18 cm BioGel A-15m column (in HBS) at 35 ml h$^{-1}$. Fractions (1 ml) were assayed for protein (Sigma bicinchoninic acid protein assay kit) and lipid (LSC) and the adsorbed protein was calculated after correction for coeluting serum protein.

*Efficacy of liposomal vincristine against ascitic P388 and solid A431 tumours*

Large unilamellar liposomes of DSPC/Chol, SM/Chol and SM/Chol/PEG$_{2000}$-PE were prepared as described above and loaded with vincristine at a vincristine–lipid ratio of 0.1:1 (w/w). For efficacy experiments with the murine ascitic P388 tumour, free and liposomal vincristine was injected i.v. into BDF1 mice that had been inoculated 24 h earlier with an i.p. injection of 10$^{6}$ P388 cells. Concentrations were adjusted to achieve vincristine doses of 2.0, 3.0 and 4.0 mg kg$^{-1}$. Animal weights and survival were followed during the subsequent 60 days. Animals surviving for 60 days were reinjected with 10$^{6}$ P388 cells to evaluate the immune component of long-term survival. Survival times (in days) for each treatment were ranked and statistically analysed using Cox's *F*-test. Comparisons indicated as having statistical significance had *P*-values from the Cox's *F*-test that were $<0.05$.

For efficacy experiments with the solid human A431 tumour (a squamous cell carcinoma), SCID mice inoculated bilaterally 14 days earlier with 2 × 10$^{6}$ A431 cells received a single i.v. injection of free vincristine or of vincristine loaded into either DSPC/Chol or SM/Chol liposomes. Concentrations were adjusted to achieve vincristine doses of 2.0 mg kg$^{-1}$. Animal weights and tumour sizes were followed during the subsequent 40 days. Tumour weight was determined by measuring tumour dimensions and calculating weight with the equation (Tomayko and Reynolds, 1989):

$$(\pi \times 6^{-1}) \times \text{length} \times \text{width} \times \text{depth}$$

### Results

### *In vitro comparison of DSPC/Chol and SM/Chol liposomes*

The chemical stability of liposomes composed of SM/Chol and DSPC/Chol was compared based on observations that hydrolysis of DSPC to MSPC and free fatty acid leads to increased vincristine leakage rates from the liposome (L Mayer, unpublished data) and on the expectation that lower hydrolysis rates should occur in SM-based lipid carriers. Liposomes of DSPC/Chol (55:45 mol/mol) or SM/Chol (55:45 mol/mol) were prepared in 0.3 M citrate buffer at pH 2.0 and incubated at 37°C in order to accelerate lipid hydrolysis and facilitate the comparison of acid-induced hydrolysis rates in these liposomes. Under these conditions, liposomes composed of SM/Chol were approximately 100-fold less susceptible to acid hydrolysis than were DSPC/Chol liposomes (Table I). Specifically, the rates of hydrolysis at 37°C and pH 2.0 were 0.090% per hour and 9.11% per hour in SM/Chol and DSPC/Chol liposomes respectively. It should be noted that, although extreme conditions (pH 2.0, 37°C) were used to accelerate the hydrolysis rates in SM/Chol and DSPC/Chol liposomes, similar results have been observed during incubation of liposomes at pH 4.0 and at various temperatures between 4°C and 37°C (data not shown).

The interactions of DSPC/Chol and SM/Chol liposomes with blood components were also compared in order to identify possible differences that might affect the biological behaviour of liposomal vincristine. The leakage of vincristine from liposomes during 6 h of incubation in plasma at 37°C (50 μg drug ml$^{-1}$ plasma) was 1.42-fold faster from DSPC/Chol liposomes than from SM/Chol liposomes (linear leakage rates of 2.7% per hour and 1.9% per hour for DSPC/Chol and SM/Chol respectively; Table I). These results correlated with observations where DSPC/Chol liposomes adsorbed 13.7 μg protein mg$^{-1}$ lipid from plasma, while no adsorbed protein could be detected on SM/Chol liposomes (detection limit of 1.75 μg protein mg$^{-1}$ lipid; Table I). Taken together, the above data suggest that SM/Chol liposomes might exhibit properties well suited for extending the circulation lifetime of encapsulated vincristine and potentially leading to a significant improvement in its therapeutic activity.

### *Lipid and drug pharmacokinetics in BDF1 mice*

We have examined the lipid and drug pharmacokinetics of DSPC/Chol and SM/Chol liposomal formulations of vincristine. These studies involved the determination of plasma clearance rates of both empty and vincristine-loaded liposomes. Plasma levels of empty DSPC/Chol and SM/Chol liposomes are shown in Figure 1. Liposomes composed of SM/Chol were removed from the circulation at a rate slower than were DSPC/Chol liposomes. Specifically, the half-lives of lipid removal from the circulation, as calculated from the slopes of the ln% lipid remaining *vs* time, were 4.0 h for DSPC/Chol liposomes and 5.7 h for SM/Chol liposomes ($r^2 \geqslant 0.996$). In liposomes loaded with vincristine, the clearance rates for both liposome types were slower, presumably because of the effect of the drug on the phagocytic activity of the RES (Mayer *et al.*, 1995). However, there was no statistical difference between clearance rates of DSPC/Chol and SM/Chol liposomes in formulations containing vincristine (Figure 2). Specifically, the half-lives for removal of vincristine-loaded liposomes from the circulation were 15.7 h for DSPC/Chol liposomes compared with 18.9 h for SM/Chol liposomes (Table II). The removal of liposomes from the circulation could be accurately described by a first-order exponential process, as indicated by the linear relationship shown in the semi-log plot of Figure 2 ($r^2 \geqslant 0.980$).

The vincristine retention characteristics of the liposomes were significantly altered by changes in the lipid composition of the vesicles. Vincristine leakage from DSPC/Chol liposomes resulted in 5.6% of the originally encapsulated vincristine remaining entrapped after 72 h in circulation (Figure 3). In contrast, vincristine leakage from SM/Chol liposomes was

**Figure 1** Amount of lipid remaining in the circulation of BDF1 mice injected with liposomes composed of DSPC/Chol (○) or SM/Chol (●). The injected dose of lipid was 20 mg kg$^{-1}$, corresponding to a total injection of approximately 430 μg of lipid. Data represent means (± s.e.) of three mice; where standard error bars are not visible, they are smaller than the size of the symbol.

**Figure 2** Amount of lipid remaining in circulation in BDF1 mice injected with large unilamellar liposomes of DSPC/Chol (○) or SM/Chol (●). Injected liposomes were loaded with vincristine at a drug–lipid ratio of approximately 0.1:1 (w/w). The injected dose of lipid was 20 mg kg$^{-1}$, corresponding to a total injection of approximately 430 μg of lipid. Data represent means (± s.e.) of three mice; where standard error bars are not visible, they are smaller than the size of the symbol.

**Table I** Summary of the *in vitro* properties of DSPC/Chol and SM/Chol liposomes

| Liposome | *Hydrolysis rate*[a] *(% h$^{-1}$)* | *Vincristine leakage*[b] *(% h$^{-1}$)* | *Protein adsorption (μg protein mg$^{-1}$ lipid)* |
|---|---|---|---|
| DSPC/Chol | 9.11 (*0.999*) | 2.7 (*0.982*) | 13.7 |
| SM/Chol | 0.090 (*0.520*) | 1.9 (*0.997*) | <1.75 |

[a]Hydrolysis at pH 2.0, 37°C ($r^2$ of the linear regression). [b]Leakage of vincristine incubated in plasma during 6 h at 37°C ($r^2$ of the linear regression).

**Table II** Plasma clearance properties of free and liposomal vincristine in BDF1 mice bearing P388 tumours

| Formulation | $t_{1/2}(r^2)^a$ L | $t_{1/2}(r^2)^a$ V | $t_{1/2}(r^2)^a$ V/L | 24 h[b] L | 24 h[b] V | 24 h[b] V/L | 72 h[b] L | 72 h[b] V | 72 h[b] V/L |
|---|---|---|---|---|---|---|---|---|---|
| Free vincristine | – | 0.16 (0.57) | – | – | 0.156 (0.035) | – | – | – | – |
| DSPC/Chol | 15.7 (0.98) | 8.0 (0.99) | 17.1 (0.99) | 160 (10) | 4.86 (0.40) | 30.3 (0.52) | 22.4 (4.3) | 0.10 (0.03) | 5.6 (2.9) |
| SM/Chol | 18.9 (0.98) | 12.1 (0.99) | 33.3 (0.99) | 186 (8.7) | 12.5 (0.52) | 67.4 (0.33) | 26.1 (7.6) | 0.66 (0.21) | 25.0 (1.1) |
| SM/Chol/$PEG_{2000}$-PE | 24.0 (0.99) | 12.7 (0.99) | 26.8 (0.99) | 243 (11) | 12.1 (0.87) | 49.7 (1.3) | 62.8 (16.5) | 0.89 (0.31) | 13.8 (1.1) |

[a]The half-lives ($t_{1/2}$), in hours, for the removal of lipid (L) and vincristine (V) from circulation and for the decrease in the vincristine–lipid ratio (V/L) were calculated from the linear regressions of the ln (μg $ml^{-1}$ plasma) *vs* time or from the ln (% vincristine/lipid remaining) *vs* time plots. The $r^2$ values for these regressions are given in brackets. [b]The levels of lipid (L) and of vincristine (V) (μg $ml^{-1}$ plasma) at 24 and 72 h after injection ( ± standard error). The vincristine–lipid ratio (V/L), expressed as a percentage of the ratio at injection, at 24 and 72 h after injection ( ± s.e.).

much slower, with 25.0% of the entrapped drug remaining in the liposomes 72 h after injection (Figure 3). The half-lives of vincristine leakage from the liposomes were 17.1 h for DSPC/Chol and 33.3 h for SM/Chol liposomes (Table II). As described for the removal of liposomes from circulation, vincristine leakage from both liposome types could be accurately described as a first-order exponential decay process. Additional increases in the retention of vincristine in SM/Chol liposomes were not observed in the presence of a larger transmembrane pH gradient ($pH_i = 2.0$, data not shown). This is in contrast to previous studies using DSPC/Chol in which a 2-fold increase in drug retention was observed when the internal buffer was changed from pH 4 to pH 2 (Boman *et al.*, 1994).

The anti-tumour efficacy of liposomal vincristine is associated with the amount of the drug remaining in circulation (Mayer *et al.*, 1993; Boman *et al.*, 1994) and, therefore, is dependent on both liposome longevity in the circulation and vincristine retention within the liposome. Free vincristine was rapidly removed from the circulation; the amount of vincristine in circulation at 4 and 24 h after injection was 30- to 80-fold lower than for liposomal vincristine (Figure 4). The total amount of vincristine remaining in the circulation was significantly lower for the liposomal DSPC/Chol formulations than for the SM/Chol formulation (Figure 4). At 72 h after i.v. injection, the amount of vincristine remaining in circulation was 0.10 μg vincristine $ml^{-1}$ plasma for DSPC/Chol compared with 0.67 μg vincristine $ml^{-1}$ plasma for SM/Chol liposomes (Figure 4). The half-life for plasma vincristine levels was 8.0 h for DSPC/Chol liposomes compared with 12.1 h for SM/Chol liposomes (Table II). The lower levels of plasma vincristine in the DSPC/Chol liposomes were primarily a consequence of the more rapid leakage of vincristine from DSPC/Chol liposomes (Figure 3).

These pharmacokinetic studies of liposomal vincristine were extended to a formulation composed of SM/Chol/$PEG_{2000}$-PE. $PEG_{2000}$-PE is a lipid known to confer increased circulation longevity on liposomes (Allen and Hansen, 1991; Allen *et al.*, 1991). A significant increase in the amount of lipid remaining in circulation was achieved by the addition of 5 mol% $PEG_{2000}$-PE to the SM/Chol mixtures. At 72 h after i.v. injection, 62.8 μg lipid $ml^{-1}$ plasma remained in circulation for SM/Chol/$PEG_{2000}$-PE liposomes compared with 26.1 μg $ml^{-1}$ plasma for SM/Chol liposomes and 22.4 μg $ml^{-1}$ plasma for DSPC/Chol liposomes (Table II). This was reflected by a significant increase in the half-life for lipid clearance from the circulation to 24.0 h for SM/Chol/$PEG_{2000}$-PE liposomes. However, the presence of 5 mol% $PEG_{2000}$-PE in SM/Chol liposomes also caused a significant increase in vincristine permeability. Specifically, only 13.8% of the entrapped vincristine remained in the liposomes after 72 h in the circulation, representing a half-life for vincristine leakage of 26.8 h for SM/Chol/$PEG_{2000}$-PE liposomes (Table II). As a consequence of the higher vincristine–lipid ratio in

**Figure 3** Vincristine–lipid ratio, expressed as a percentage of the injected ratio, in the plasma of BDF1 mice at various times after the injection of large unilamellar liposomes of DSPC/Chol (○) or SM/Chol (●). Mice were injected with liposomes at a vincristine–lipid ratio of approximately 0.1:1, corresponding to a lipid dose of 20 mg $kg^{-1}$ and a vincristine dose of 2.0 mg $kg^{-1}$. Total amounts injected were approximately 430 μg of lipid and 43 μg of vincristine. Data represent means (± s.e.) of three mice; where standard error bars are not visible, they are smaller than the size of the symbol.

**Figure 4** Total vincristine remaining in the plasma of BDF1 mice at various times after i.v. administration of free vincristine (□) or of large unilamellar liposomes of DSPC/Chol (○) or SM/Chol (●) at a vincristine–lipid ratio of 0.1:1 (w/w), corresponding to a lipid dose of 20 mg $kg^{-1}$ and a vincristine dose of 2.0 mg $kg^{-1}$. Total amounts injected were approximately 430 μg of lipid and 43 μg of vincristine. Data represent means ( ± standard error) of three mice; where standard error bars are not visible, they are smaller than the size of the symbol.

SM/Chol liposomes, compared with SM/Chol/$PEG_{2000}$-PE liposomes, and the more rapid removal of SM/Chol liposomes from circulation than for SM/Chol/$PEG_{2000}$-PE liposomes, both formulations had equal amounts of vincristine remaining in the circulation at all times between 4 h and 72 h after i.v. injection. Specifically, the half-lives for removal of vincristine from the circulation were 12.7 h for SM/Chol/$PEG_{2000}$-PE, compared with 12.1 h for SM/Chol liposomes (Table II). Therefore, we have observed no overall improvement in vincristine circulation longevity *in vivo* by the addition of $PEG_{2000}$-PE to SM/Chol liposomes.

*Vincristine accumulation in the site of P388 ascitic tumour growth*

In order to determine if the pharmacokinetic characteristics observed for DSPC/Chol and SM/Chol in plasma affected the tendency for vincristine and lipid to accumulate in an extravascular site, we examined the accumulation of lipid and drug in the peritoneal cavity of mice inoculated 24 h earlier with P388 cells. No significant difference was observed in the accumulation of lipid in the peritoneal cavity after administration of DSPC/Chol or SM/Chol liposomes (data not shown). This result suggests that there was little or no difference between these liposome formulations in their abilities to extravasate to the site of the peritoneal P388 tumour cell inoculation. The amount of free vincristine accumulating in the peritoneal cavity was highest (875 ng) at 7 min after injection of the free drug and decreased to levels less than 217 ng within 4 h (Figure 5). The accumulation of vincristine from DSPC/Chol liposomes in the peritoneal cavity peaked at 628 ng vincristine per lavage at 4 h after liposome injection and decreased to less than 188 ng vincristine per lavage at times in the range between 24 and 72 h (Figure 5). In contrast, vincristine from SM/Chol formulations showed sustained delivery of vincristine for up to 24–48 h after liposome injection (Figure 5). Specifically, SM/Chol formulations of vincristine delivered 889–605 ng of vincristine per lavage over the time period between 4 and 24 h after injection. Vincristine accumulation in the peritoneal cavity from SM/Chol did not decrease to levels equivalent to those from DSPC/Chol liposomes until 48 h after injection. The total exposure of P388 cells residing in the peritoneal cavity to vincristine delivered by SM/Chol liposomes was approximately 30% greater than that achieved by DSPC/Chol liposomes. In addition, the accumulation of vincristine from liposomes of SM/Chol/$PEG_{2000}$-PE into the peritoneal cavity of mice inoculated with P388 cells was identical to that observed for SM/Chol liposomes (data not shown).

The vincristine–lipid ratios observed in both DSPC/Chol and SM/Chol liposomes that had extravasated from the circulation to the peritoneal cavity were similar to those observed for liposomes remaining in the plasma. Specifically, the vincristine–lipid ratios observed for SM/Chol liposomes in the peritoneum were much greater than those for peritoneal DSPC/Chol liposomes (Figure 6). In addition, the peritoneal vincristine–lipid ratios were similar to those observed in the plasma for both liposomal formulations (Figure 6). These results suggest that the increased delivery of vincristine to the peritoneal cavity by SM/Chol formulations of vincristine, compared with DSPC/Chol formulations, was not a consequence of delivery of free vincristine that had leaked from liposomes in plasma. Rather, these data suggest that both SM/Chol and DSPC/Chol liposomes, containing vincristine, were extravasating from the circulation and accumulating at the site where the P388 cells were residing, as observed previously in similar systems (Mayer *et al*., 1995).

*Efficacy of liposomal vincristine formulations against P388 tumours*

Therapeutic studies were undertaken to determine if the increased delivery of vincristine to the site of P388 tumour growth observed for SM/Chol liposomes (Figure 5) resulted in an increase in anti-tumour activity. BDF1 mice bearing P388 tumours were untreated or were treated with free vincristine or liposomal vincristine formulations of DSPC/Chol or SM/Chol at a drug–lipid ratio of 0.1:1.0 (w/w). For free vincristine at 2.0, 3.0 and 4.0 mg $kg^{-1}$, a significant increase in survival was observed compared with controls. Specifically, median survival increased from 11.0 days to 16–16.5 days, representing increase in lifespan (ILS) values of 33–38% (Table III and Figure 7).

At a vincristine dose of 2 mg $kg^{-1}$, the DSPC/Chol formulation increased the median survival to 26.5 days (ILS = 141% compared with control mice receiving no treatment), however, the SM/Chol formulation was significantly more effective ($P<0.05$) than DSPC/Chol, with 50% of the SM/Chol treatment group surviving at 60 days after administration of the P388 tumours (median survival > 48.5 days, ILS > 341%; Figure 7 and Table III). The greatest anti-tumour efficacy of the liposomal formulations of vincristine was observed at the 3 mg $kg^{-1}$ vincristine dose, at which SM/Chol-entrapped vincristine effected a 90% survival rate (ILS > 445% and median survival > 60 days; Table III). The SM/Chol formulation at 3 mg $kg^{-1}$ vincristine had a survival curve that was significantly different ($P<0.05$) from the DSPC/Chol formulation (Figure 7 and Table III). At a vincristine dose of 4 mg $kg^{-1}$, both formulations had 40–50% survival at 60 days after inoculation of the P388 tumour, however at this vincristine dose the survival curves for the

**Figure 5** Accumulation of vincristine in the peritoneal cavity of BDF1 mice bearing peritoneal P388 tumour cells after i.v. administration of free vincristine (□) or of large unilamellar liposomes of DSPC/Chol (○) or SM/Chol (●) containing vincristine at a drug–lipid ratio of 0.1:1 and a vincristine dosage of 2.0 mg $kg^{-1}$. Data represent means (± s.e.) of four mice; where standard error bars are not visible, they are smaller than the size of the symbol.

**Figure 6** Comparison of the vincristine–lipid ratio (expressed as a percentage of the injected ratio) between liposomes remaining in circulation (●, ○) and those extravasated to the peritoneal cavity (■, □) for DSPC/Chol (○, □) and SM/Chol (●, ■) liposomes. Data for the vincristine–lipid ratios for liposomes in plasma were taken from Figure 3 for comparison.

**Table III** Anti-tumour efficacy of free and liposomal formulations of vincristine in BDF1 mice bearing P388 tumours

| *Drug dose[a] (mg kg$^{-1}$)* | *Treatment* | *Maximum per cent weight change (day)* | *Median survival (days)* | *ILS[b] (%)* | *Sixty-day survival* |
|---|---|---|---|---|---|
| 0 (control) | – | + 11.8 (7) | 11.0 | – | 0/15[c] |
| 2.0 | Free vincristine | – 3.4 (5) | 16.0 | 33 | 0/10 |
| | DSPC/Chol | – 12.3 (4) | 26.5 | 141 | 1/10[d] |
| | SM/Chol | – 10.9 (3) | >48.5 | >341 | 5/10[d] |
| 3.0 | Free vincristine | – 9.0 (5) | 16.5 | 38 | 0/10 |
| | DSPC/Chol | – 14.6 (4) | 34.5 | 214 | 3/10[e] |
| | SM/Chol | – 14.9 (4) | >60.0 | >445 | 9/10[e] |
| 4.0 | Free vincristine | – 15.5 (5) | 16.5 | 38 | 0/10 |
| | DSPC/Chol | – 20.2 (5) | 34.0 | 209 | 4/10 |
| | SM/Chol | – 18.4 (4) | >47.5 | >332 | 5/10 |

[a]Lipid dose was 10-fold greater than the drug dose. [b]ILS (increase in lifespan) was calculated from the median survival times of treated and control animals. [c]All treatment groups were significantly different from the controls at $P<0.05$. [d]At 2.0 mg kg$^{-1}$ vincristine, DSPC/Chol was significantly different from SM/Chol at $P<0.05$. [e]At 3.0 mg kg$^{-1}$ vincristine, DSPC/Chol was significantly different from SM/Chol at $P<0.05$.

different liposomal formulations were not significantly different. Further, at 4 mg kg$^{-1}$ vincristine, several deaths occurred in the treated animals before the earliest of the tumour-related deaths in control animals (days 6–9; Figure 7 and Table III). These deaths were consistent with vincristine toxicity, rather than the results of excessive tumour growth (Figure 7). There was no indication that SM/Chol formulations were significantly more toxic than were the DSPC/Chol formulations based on the nadir animal weights following treatment (Table III). It should also be added that the anti-tumour efficacy of SM/Chol/PEG$_{2000}$-PE formulations containing vincristine were not significantly different from those observed for the SM/Chol formulations at all doses examined (data not shown). Further, administration of empty liposomes of SM/Chol at doses of either 40 or 80 mg kg$^{-1}$ lipid had no therapeutic benefit (data not shown).

*Pharmacokinetics, tumour loading and therapy in SCID mice bearing A431 tumours*

Tumour loading and anti-tumour efficacy properties of DSPC/Chol and SM/Chol liposomal formulations of vincristine were also determined in mice bearing solid human A431 squamous cell xenograft tumours. These experiments were undertaken to ensure that the positive results observed in the murine ascitic P388 tumour model were representative of other tumour types. SCID mice bearing 100–200 mg solid human A431 tumours were injected i.v. with free vincristine or with liposomes of either DSPC/Chol or SM/Chol containing vincristine. Encapsulation of vincristine in DSPC/Chol and SM/Chol liposomes increased the amount of vincristine remaining in circulation 24 h after administration by 28- and 87-fold respectively compared with free vincristine (Figure 8a). As observed in BDF1 mice bearing P388 tumours, the amount of vincristine remaining in the circulation in SM/Chol liposomes at 24 h after injection was approximately 3-fold greater than for vincristine encapsulated in DSPC/Chol liposomes (Figure 8a).

Improved vincristine circulation longevity correlated with increases in the loading of vincristine in the A431 tumours (Figure 8b). Specifically, free vincristine levels in A431 tumours were highest (0.856 μg g$^{-1}$ tumour) at 0.5 h after injection and decreased to 0.32 μg g$^{-1}$ tumour at 24 h (Figure 8b). Encapsulation of vincristine in DSPC/Chol liposomes increased the amount of vincristine present in A431 tumours at 4–48 h after administration to 1.3–1.55 μg g$^{-1}$ tumour respectively (Figure 8b). Encapsulation of vincristine in SM/Chol liposomes resulted in a further increase in vincristine delivery to A431 tumours at 24–48 h after injection to 2.8–3.2 μg g$^{-1}$ tumour, representing a 2-fold increase in the delivery obtained with DSPC/Chol liposomes. As observed in the murine ascitic tumour model, the vincristine–lipid ratios observed in the solid human A431 tumours were very similar to those observed in the plasma. That is, for vincristine

**Figure 7** Anti-tumour efficacy of free vincristine and liposomal formulations of vincristine. BDF1 mice bearing P388 tumours were untreated (■) or were injected with free vincristine (□) or large unilamellar liposomes of DSPC/Chol (○) or SM/Chol (●) containing vincristine at a drug–lipid ratio of 0.1:1 (w/w). Concentrations were adjusted before injection to achieve vincristine doses of 2.0 (**a**), 3.0 (**b**) and 4.0 (**c**) mg kg$^{-1}$.

encapsulated in DSPC/Chol liposomes, the vincristine–lipid (w/w) ratios at 24 h after injection were 0.022 in the plasma and 0.029 in the tumour, while for vincristine encapsulated in

**Figure 8** Plasma (a) and tumour (b) levels of vincristine after administration of free and liposomal vincristine in SCID mice bearing A431 tumours. SCID mice bearing two A431 tumours were injected i.v. with free vincristine (□) or with large unilamellar liposomes of DSPC/Chol (○) or SM/Chol (●) containing vincristine at a drug–lipid ratio of 0.1:1 (w/w). Vincristine was injected at a dose of 2.0 mg $kg^{-1}$, representing a lipid dose of 20 mg $kg^{-1}$. Data represent means (± s.e.) of three mice (six tumours); where standard error bars are not visible, they are smaller than the size of the symbol.

**Figure 9** Anti-tumour efficacy of free and liposomal vincristine in SCID mice bearing A431 tumours. SCID mice bearing two A431 tumours received no treatment (■) or were injected i.v. with free vincristine (□) or with large unilamellar liposomes of DSPC/Chol (○) or SM/Chol (●) containing vincristine at a drug–lipid ratio of 0.1:1 (w/w). Vincristine was injected at a dose of 2.0 mg $kg^{-1}$, representing a lipid dose of 20 mg $kg^{-1}$. Data represent the weight of A431 tumours (expressed as a percentage of the tumour weight immediately before treatment) and are the means (± s.e.) of 8–10 tumours in 4–5 mice.

SM/Chol liposomes the vincristine–lipid ratios were 0.055 in the plasma and 0.050 in the tumour.

The anti-tumour efficacy of free and liposomal vincristine against A431 was closely correlated with vincristine accumulation at the tumour site (Figure 9). SCID mice bearing the A431 tumours that received no treatment showed a 100% increase in tumour weight within 4–5 days after treatment was initiated and required termination within 10 days when the tumour exceeded 10% of the total body weight. Tumour-bearing SCID mice treated with free vincristine at 2.0 mg $kg^{-1}$ had a brief delay in tumour growth (100% increase in tumour weight achieved within 6–8 days) but required termination between 10 and 12 days. In contrast, treatment with vincristine encapsulated in DSPC/Chol liposomes resulted in a significant delay in tumour growth (100% increase in tumour weight at 15–20 days, termination at 21 days after treatment). This therapy was further enhanced by a single treatment of vincristine encapsulated in SM/Chol liposomes. In this treatment group a small but consistent decrease in tumour size was observed. At 15 days after injection, several tumours were palpable but unmeasurable, and by 33 days after treatment several tumours were not palpable. Of the five mice (total of ten tumours) treated with SM/Chol liposomal vincristine, one animal was killed early because of tumour ulceration, not due to tumour growth. Of the eight tumours remaining at 40 days after liposome injection, histological analysis indicated that all eight were actively dividing squamous cell carcinomas of a mass undetectable by physical examination. Therefore, treatment with SM/Chol liposomal vincristine effected a significant reduction in tumour growth, although none of the original tumours were cured.

### Discussion

Several reports have documented the ability of a variety of liposomes to decrease the toxicity and/or improve the efficacy of vincristine (Layton and Trouet, 1980; Mayer *et al.*, 1990*a*, 1993, 1995; Vaage *et al.*, 1993; Boman *et al.*, 1994). Early studies revealed that vincristine is relatively permeable to the phospholipid membranes, a characteristic that limits the use of lipids containing unsaturated fatty acids, such as egg PC (Mayer *et al.*, 1990*a*). Subsequent investigations (Mayer *et al.*, 1993, 1995; Boman *et al.*, 1994) demonstrated that the therapeutic activity of liposomal vincristine correlates with the drug circulation lifetime and results from drug carried directly to the tumour site within the lipid vehicle. Consequently, liposomes that rapidly release entrapped vincristine (i.e. egg PC liposomes) or are rapidly cleared by the reticulo endothelial system after i.v. administration have only modest pharmacological improvements over free vincristine (Mayer *et al.*, 1993). These observations initiated attempts to improve vincristine retention through the use of larger transmembrane pH gradients and/or the presence of $GM_1$ (Boman *et al.*, 1994). While these alterations optimised the therapeutic properties of liposomal vincristine, they also compromised the clinical utility of the formulations owing to problems associated with the use of $GM_1$ in human applications and with the high rate of phospholipid hydrolysis and degradation of the liposomes in low-pH buffers. We therefore examined the *in vitro* and *in vivo* properties of liposomal formulations of vincristine that are based on sphingomyelin rather than on phosphatidylcholine. This approach was based on the possibility that the amide-linked aliphatic chain of sphingomyelin would be more resistant to chemical and biological degradation than would be the ester-linked acyl chains of phosphatidylcholine (Table I).

Empty liposomes composed of SM/Chol displayed circulation lifetimes that were increased compared with DSPC/Chol liposomes (Figure 1). The extended circulation times for empty SM/Chol liposomes is consistent with the lower adsorption of serum proteins to SM/Chol liposomes. An inverse relationship between protein opsonisation to liposomes and circulation longevity has been reported previously (Senior *et al.*, 1985; Hwang, 1987; Chonn *et al.*, 1992) and is consistent with the importance of protein–liposome interaction in liposome pharmacokinetics. Extended circulation lifetimes for SM/Chol liposomes have also been observed by other workers (Allen and Chonn, 1987; Allen *et al.*, 1989). However, it should be noted that previous reports employed an entrapped aqueous solute ($^{125}$I-labelled tyraminylinulin) as a marker for liposome distribution. Consequently, it is possible that the apparent increase in liposome longevity in the presence of sphingomyelin (Allen and Chonn, 1987; Allen *et al.*, 1989) arose in part from increased solute retention by sphingomyelin. In vincristine-loaded liposomes, we observed no significant difference in circulation longevity between DSPC/Chol and SM/Chol formulations (Figure 2). The apparent inconsistency between the relative circulation lifetimes observed for empty and vincristine-loaded liposomes of DSPC/Chol and SM/Chol may be related to the 'blockade' effect of liposomes containing cytotoxic agents (Bally *et al.*, 1990; Parr *et al.*, 1993). Vincristine released from DSPC/Chol liposomes within macrophages may reduce the ability of these cells to further remove liposomes from the circulation.

SM/Chol liposomes had significantly improved vincristine retention characteristics compared with DSPC/Chol liposomes (Figure 3). The effect of sphingomyelin on vincristine retention resulted in a 6- to 7-fold increase in plasma levels of vincristine between 48 and 72 h after i.v. administration in BDF1 mice compared with the DSPC/Chol formulation of vincristine (Figure 4). The increased levels of vincristine remaining in circulation in the plasma using SM/Chol formulations (Figures 4 and 8a) were correlated with greater amounts of vincristine delivered to the site of P388 tumour cell inoculation (the peritoneal cavity; Figure 5) and to subcutaneous A431 tumours grown in SCID mice (Figure 8b) as well as with increased efficacy against these tumours (Figures 7 and 9 and Table III). At vincristine doses of 2.0–3.0 mg $kg^{-1}$, SM/Chol formulations of vincristine were significantly more effective than free vincristine or vincristine encapsulated in DSPC/Chol formulations at reducing P388 and A431 tumour progression.

Recent studies on the relationship between vincristine pharmacokinetics and therapeutic activity have demonstrated that the increased drug accumulation in tumours and increased anti-tumour activity observed for vincristine encapsulated in DSPC/Chol liposomes is a consequence of the ability of the liposomes to deliver encapsulated vincristine directly to the tumour site (Mayer *et al.*, 1995). This is consistent with previous studies which have shown that the delivery of a liposomal drug to a tumour is associated with extravasation of liposomes to the interstitial, or extravascular, spaces of the tumours (Gabizon, 1992, 1994; Bally *et al.*, 1994; Yuan *et al.*, 1994). Our analysis reported here is consistent with these studies and suggests that the increased delivery of vincristine to tumours by SM/Chol formulations was the consequence of liposome extravasation into the tumour and was not due to tumour uptake of free drug that had been released from liposomes in the circulation. Specifically, although vincristine levels in the tumours were greater for SM/Chol formulations than for DSPC/Chol formulations, the accumulation of liposomes in the tumours was similar for both systems. The fact that the vincristine–lipid ratios in the tumours correlated very closely with vincristine–lipid ratios in the plasma for both liposomal formulations indicates that vincristine retention by the lipid carrier is a key feature affecting both tumour exposure to liposomal vincristine and the anti-tumour efficacy of liposomal vincristine. It should be noted that, while extravasation to the peritoneum is dependent on pre-existing vasculature, extravasation to solid tumours occurs after the formation of new tumour capillaries. Therefore, extravasation of liposomal drug delivery vehicles to peritoneal and solid tumours may not be directly comparable. The relative contribution of passive liposome extravasation via a pore-leakage mechanism *vs* liposome extravasation mediated through a cell-dependent mechanism (transcytosis) is under active investigation.

The pharmacokinetic and therapeutic results presented here are very similar to those reported recently by Boman *et al.* (1994), who found that the presence of the ganglioside $GM_1$ in DSPC/Chol liposomes with an intraliposomal pH of 2.0 significantly increased *in vivo* vincristine retention within the liposomes and effected a significant increase in efficacy against the P388 tumour. The results presented here demonstrate that a formulation with similar pharmacokinetic and therapeutic characteristics can be obtained with a relatively simple mixture composed of sphingomyelin and cholesterol. This formulation does not require either extreme intraliposomal pH conditions or the addition of lipids that confer extended circulation longevity. In fact, the pharmacokinetic and tumour accumulation results suggest that SM/Chol liposomes possess characteristics that extend the circulation longevity of liposomes. These characteristics appear to be related to the reduced interaction of SM/Chol liposomes with both phagocytic cells (M Webb, unpublished observation) and serum proteins (Table I), similar to the behaviour of liposomes that contain $GM_1$ (Chonn *et al.*, 1992) or PEG-PE (Blume and Cevc, 1993). It is interesting that the addition of $PEG_{2000}$-PE to SM/Chol liposomes resulted in the expected increase in liposome circulation longevity, however $PEG_{2000}$-PE also caused a significant increase in the leakage of vincristine from the liposomes. The net result of these effects was no enhancement of vincristine circulation longevity (Table II). Further, the results presented here indicated that vincristine accumulation in the peritoneal cavity (an extravascular site) and anti-tumour activity against a peritoneal P388 tumour were not affected by the addition of $PEG_{2000}$-PE to SM/Chol liposomes. The data presented here question the pharmacokinetic and therapeutic benefits obtained through the use of sterically stabilised lipids for relatively membrane-permeable agents such as vincristine.

It should be noted that several reports have suggested that sphingomyelin-containing liposomes are more toxic than PC-containing liposomes (Weereratne *et al.*, 1983; Allen *et al.*, 1984; Allen and Smuckler, 1985). However, these studies, in which mice received high-dose chronic injections (10 i.p. injections during a 20 day period) of liposomes (Weereratne *et al.*, 1983), found that small unilamellar vesicles containing sphingomyelin were not different from either controls or similar PC-containing liposomes (Allen *et al.*, 1984). In addition, the observed effects (liver granulomas) also occurred in mice receiving injections of DSPC/cholesterol liposomes (Allen and Smuckler, 1985). Our data (Table III) suggest that vincristine formulated in SM/Chol liposomes was not significantly more toxic than vincristine in DSPC/Chol liposomes. While weight loss associated with the administration of liposomal vincristine was somewhat greater than that incurred for free vincristine (Table III), animals receiving free drug also had more rapid tumour growth, and weight gain than did the animals receiving liposomal drug. A more detailed analysis of the toxicities associated with single and multiple i.v. administrations has recently shown that liposomal vincristine is less toxic than the free drug in mice and has very similar toxicity to the free drug in beagle dogs (Kanter *et al.*, 1994). Overall, these results suggest that the therapeutic index of SM/Chol liposomal vincristine is significantly better than either free vincristine or vincristine encapsulated in DSPC/Chol liposomes.

In summary, we have demonstrated that liposomal formulations of vincristine based on sphingomyelin/cholesterol have several significant advantages over those based on DSPC/cholesterol. These liposomal formulations are more resistant to chemical degradation and drug release *in vitro*. Further, they also have improved pharmacokinetic and therapeutic characteristics. Specifically, the anti-tumour activity of vincristine can be optimised from a drug that in free form has only minimal activity against P388 ascitic tumours and A431 human solid tumours to a drug that causes cures and significant increases in lifespan. The ability to achieve these effects without the use of unusual lipids such as $GM_1$ or sterically stabilised lipids and/or extreme pH conditions increases the likelihood that this technology will be of use for applications in a variety of human neoplasms.

#### Abbreviations

Chol, cholesterol; DSPC, distearoylphosphatidylcholine; PC, phosphatidylcholine: MSPC, monostearoylphosphatidylcholine; $PEG_{2000}$-PE, polyethyleneglycol$_{2000}$-distearoylphosphatidylethanolamine; RES, reticuloendothelial system; SCID, severe combined immune deficiency; SM, egg sphingomyelin.

**Acknowledgements**

This work was supported by an operating grant from the Cancer Research Society Inc. of Canada and from Inex Pharmaceuticals Corporation, Vancouver. The authors wish to thank Ms Jean Heggie and Ms Ginette St. Onge for their technical assistance.

### References

ALLEN TM AND CHONN A. (1987). Large unilamellar liposomes with low uptake into the reticuloendothelial system. *FEBS Lett.*, **223**, 42–46.

ALLEN TM AND HANSEN C. (1991). Pharmacokinetics of stealth versus conventional liposomes: effect of dose. *Biochim. Biophys. Acta*, **1068**, 133–141.

ALLEN TM AND SMUCKLER EA. (1985). Liver pathology accompanying chronic liposome administration in mouse. *Res. Commun. Chem. Pathol. Pharmacol.*, **50**, 281–290.

ALLEN TM, MURRAY L, MACKEIGAN S AND SHAH M. (1984). Chronic liposome administration in mice: effects on reticuloendothelial function and tissue distribution. *J. Pharmacol Exp. Ther.*, **229**, 267–275.

ALLEN TM, HANSEN C AND RUTLEDGE J. (1989). Liposomes with prolonged circulation times: factors affecting uptake by reticuloendothelial and other tissues. *Biochim. Biophys. Acta*, **981**, 27–35.

ALLEN TM, HANSEN C, MARTIN F, REDEMANN C AND YAU-YOUNG A. (1991). Liposomes containing synthetic lipid derivatives of poly(ethylene glycol) show prolonged circulation half-lives in vivo. *Biochim. Biophys. Acta*, **1066**, 29–36.

BALLY MB, NAYAR R, MASIN D, HOPE MJ, CULLIS PR AND MAYER LD. (1990). Liposomes with entrapped doxorubicin exhibit extended blood residence times. *Biochim. Biophys. Acta*, **1023**, 113–139.

BALLY MB, MASIN D, NAYAR R, CULLIS PR AND MAYER LD. (1994). Transfer of liposomal drug carriers from the blood to the peritoneal cavity of normal and ascitic tumour-bearing mice. *Cancer Chemother. Pharmacol.*, **34**, 137–146.

BARTLETT GR. (1959). Phosphorus assay in column chromatography. *J. Biol. Chem.*, **234**, 466–468.

BLUME G AND CEVC G. (1993). Molecular mechanism of the lipid vesicle longevity in vivo. *Biochim. Biophys. Acta*, **1146**, 157–168.

BOMAN L, MASIN D, MAYER LD, CULLIS PR AND BALLY MB. (1994). A liposomal vincristine preparation which exhibits increased drug retention and increased circulation longevity cures mice bearing P388 tumours. *Cancer Res.*, **54**, 2830–2833.

CHONN A, SEMPLE SC AND CULLIS PR. (1992). Association of blood proteins with large unilamellar liposomes *in vivo*. *J. Biol. Chem.*, **267**, 18759–18765.

GABIZON AA. (1992). Selective tumour localization and improved therapeutic index of anthracyclines encapsulated in long-circulating liposomes. *Cancer Res.*, **52**, 891–896.

GABIZON A, KATANE R, UZIELY B, KAUFMAN B, SAFRA T, COHEN R, MARTIN F, HUANG A AND BARENHOLZ Y. (1994). Prolonged circulation time and enhanced accumulation in malignant exudates of doxorubicin encapsulated in polyethylene-glycol coated liposomes. *Cancer Res.*, **54**, 987–992.

HORTON JK, HOUGHTON PJ AND HOUGHTON JA. (1988). Relationships between tumour responsiveness, vincristine pharmacokinetics and arrest of mitosis in human tumour xenografts. *Biochem. Pharmacol.*, **37**, 3995–4000.

HWANG KJ. (1987). Liposome pharmacokinetics. In *Liposomes: From Biophysics to Therapeutics*, Ostro, MJ. (ed.) pp. 109–156. Marcel Dekker: New York.

KANTER PM, KLAICH GM, BULLARD GA, KING JM, BALLY MB AND MAYER LD. (1994). Liposome encapsulated vincristine: preclinical toxicologic and pharmacologic comparison with free vincristine and empty liposomes in mice, rats and dogs. *Anti-Cancer Drugs*, **5**, 579–590.

LAYTON D AND TROUET A. (1990). A comparison of the therapeutic effects of free and liposomally encapsulated vincristine in leukemia mice. *Eur. J. Cancer*, **16**, 949–950.

MAYER LD, HOPE MJ, CULLIS PR AND JANOFF AS. (1985). Solute distributions and trapping efficiencies observed in freeze-thawed multilamellar vesicles. *Biochim. Biophys. Acta*, **817**, 193–196.

MAYER LD, BALLY MB, LOUGHREY H, MASIN D AND CULLIS PR. (1990*a*). Liposomal vincristine preparations which exhibit decreased drug toxicity and increased activity against murine L1210 and P388 tumours. *Cancer Res.*, **50**, 575–579.

MAYER LD, TAI LCL, BALLY MB, MITILENES GN, GINSBERG RS AND CULLIS PR. (1990*b*). Characterization of liposomal systems containing doxorubicin entrapped in response to pH gradients. *Biochim. Biophys. Acta*, **1025**, 143–151.

MAYER LD, NAYAR R, THIES RL, BOMAN NL, CULLIS PR AND BALLY MB. (1993). Identification of vesicle properties that enhance the antitumour activity of liposomal vincristine against murine L1210 leukemia. *Cancer Chemother. Pharmacol.*, **33**, 17–24.

MAYER LD, MASIN D, NAYAR R, BOMAN NL AND BALLY MB. (1995). Pharmacology of liposomal vincristine in mice bearing L1210 ascitic and B16/BL6 solid tumours. *Br. J. Cancer*, **71**, 482–488.

PARR MJ, BALLY MB AND CULLIS PR. (1993). The presence of $G_{M1}$ in liposomes with entrapped doxorubicin does not prevent RES blockade. *Biochim. Biophys. Acta*, **1168**, 249–252.

SENIOR J, CRAWLEY JCW AND GREGORIADIS G. (1985). Tissue distribution of liposomes exhibiting long half-lives in the circulation after intravenous injection. *Biochim. Biophys. Acta*, **839**, 1–8.

TOMAYKO MM AND REYNOLDS CP. (1989). Determination of subcutaneous tumour size in athymic (nude) mice. *Cancer Chemother. Pharmacol.*, **24**, 148–155.

VAAGE J, DONOVAN D, MAYHEW E, USTER P AND WOODLE M. (1993). Therapy of mouse mammary carcinomas with vincristine and doxorubicin encapsulated in sterically stabilized liposomes. *Int. J. Cancer*, **54**, 959–964.

WEERERATNE EAH, GREGORIADIS G AND CROW J. (1983). Toxicity of sphingomyelin-containing liposomes after chronic injection into mice. *Br. J. Exp. Pathol.*, **64**, 670–676.

YUAN F, LEUNIG M, HUANG SK, BERK DA, PAPAHADJOPOULOS D AND JANE RK. (1994). Microvascular permeability and interstitial penetration of stearically stabilized 'Stealth' liposomes in a human tumour xenograft. *Cancer Res.*, **54**, 3352–3356.